# Lower ANXA3 Levels May Be Related to Major Depressive Disorder

**DOI:** 10.3390/life15091456

**Published:** 2025-09-17

**Authors:** Mine Büşra Bozkürk, Kadir Özdel, Bilge Ozan Çiçek, Özkan Önder, Selin Yıldız, Alpaslan Öztürk

**Affiliations:** 1 Ankara Etlik City Hospital, Department of Clinical Biochemistry, 06170 Ankara, Türkiye; bilgeozancicek@hotmail.com (B.O.Ç.); onderozkan13@hotmail.com (Ö.Ö.); slnnnyldzzz89@gmail.com (S.Y.); dralpaslanozturk@gmail.com (A.Ö.); 2 Ankara Etlik City Hospital, Department of Psychiatry, 06170 Ankara, Türkiye; kadir.ozdel@sbu.edu.tr

**Keywords:** Annexin A3, biomarker, depression severity, major depressive disorder

## Abstract

Annexin A3 (ANXA3) is a calcium-binding protein that plays a role in membrane phospholipid metabolism and inflammation, and significant alterations have been shown in some psychotic disorders. Because its association with major depressive disorder (MDD) is unclear, we aimed to compare serum ANXA3 levels in patients with MDD and healthy controls and to investigate their relationship with depression severity. Serum ANXA3 concentrations in 90 patients diagnosed with MDD and 90 healthy controls were measured by ELISA. Depression severity was assessed using the Beck Depression Inventory (BDI). Serum ANXA3 levels were significantly lower in patients with MDD compared to controls (*p* < 0.001). ANXA3 showed negative correlations with neutrophil count, platelet count, neutrophil-to-lymphocyte ratio (NLR), glucose, creatinine, and ALT levels, and positive correlations with lymphocyte and red blood cell counts. We found that low ANXA3 levels, high NLR, and glucose dysregulation predicted greater depression severity. Using ROC analysis, we demonstrated that ANXA3 has high discriminatory power in distinguishing moderate to severe cases of MDD (AUC > 0.90). ANXA3 may serve as a biomarker of depression severity. Further studies are needed to clarify its clinical utility and confirm whether ANXA3 alterations represent state or trait markers of depression.

## 1. Introduction

Annexins are a large family of calcium-binding proteins found in various tissues and cell types [1]. Annexins interact with calcium and phospholipids found in all biological membranes, potentially playing important roles in membrane biology [2]. Calcium binding has been suggested to underlie the effects of annexins in processes such as anticoagulation, endocytosis, exocytosis, signal transduction, cellular proliferation, and apoptosis [3,4]. Mammals possess 12 different annexins, ranging from A1 to A11 and A13 [5]. Alterations in the tissue or cellular expression of annexins are associated with a wide variety of pathological conditions, including asthma [6], atherosclerosis [7], autoimmune diseases [8], malignancies [9], Parkinson’s disease [10], and Alzheimer’s disease [11]. Annexin A3 (ANXA3) has important functions in widespread activity and membrane phospholipid metabolism [12,13], and these functions have been shown to play a role in the pathogenesis of psychotic disorders [14]. A significant study in 2019 reported that T-type calcium channels could potentially be a good target for anxiety treatment. This study evaluated that excessive activation of these channels in stressful situations could cause anxiety; therefore, blocking these channels during stressful situations could alleviate anxiety [15].

In vitro studies have examined the possible interactions between benzodiazepines and annexins. Some studies have demonstrated that annexins can bind to 1,4-benzodiazepines with high affinity and can, therefore, be considered benzodiazepine receptors [16].

In a 2021 study published in the journal Neural Plasticity, “Annexin A3 as a Marker Protein for Microglia in the Central Nervous System of Rats,” the expression of ANXA3 in microglia was investigated [17]. This study showed that ANXA3-positive cells were abundant and evenly distributed throughout the brain tissue and spinal cord of the adult rats. The morphology and distribution of ANXA3-labeled microglia were found to be quite similar to those labeled with the microglial-specific markers CD11b and Iba1 in the central nervous system. ANXA3 knockdown inhibited microglial proliferation and migration, whereas ANXA3 overexpression increased these activities. In conclusion, this study suggests that ANXA3 may be a novel marker for parenchymal microglia in the central nervous system of adult rats and may have physiological functions.

Despite increasing evidence regarding the importance of annexin types in psychiatric disorders, serum ANXA3 levels have not been investigated in patients with major depressive disorder (MDD) and their relationship to depression severity has not been examined. Therefore, our study aimed to fill this gap by comparing serum ANXA3 concentrations in MDD patients with healthy controls and analyzing their relationship to the severity of depressive symptoms.

## 2. Materials and Methods

### 2.1. Study Setting and Study Population

In our study, 90 patients diagnosed with depression according to the Diagnostic and Statistical Manual of Mental Disorders (DSM-V) and SCID-5-CV (the Structured Clinical Interview for DSM-5 Disorders: Clinician Version) criteria were enrolled in the psychiatry outpatient clinics of the Etlik City Hospital. A healthy control group of 90 individuals was included for comparison with the patient group. The healthy control group consisted of individuals who were not diagnosed with any chronic disease (asthma, chronic cardiovascular disease, neurodegenerative disease (Alzheimer’s, Parkinson’s, and Pick’s disease), demyelinating disease (such as multiple sclerosis), autoimmune diseases), cancer, or psychiatric disease, were not taking medication, and had normal laboratory values. The presence of these features was also applied as exclusion criteria in the patient group. The healthy control and patient groups were matched for age and sex. The complete blood count (CBC) parameters, biochemical parameters, and ANXA3 levels were compared between the groups. Additionally, those in the major depression group completed the Beck Depression Inventory (BDI), which assessed the severity of illness. Thus, major depression levels were determined. These levels were compared with those of ANXA3.

The study was conducted in accordance with the Declaration of Helsinki, and the protocol was approved by the Etlik City Hospital Ethics Committee (dated: 2 July 2025; acceptance Number: 2025-040).

### 2.2. ELISA and Laboratory Analysis

Venous blood samples were collected from all participants included in the study. Serum samples were obtained by centrifugation (3000–3500 rpm for 15 min). Serum samples remaining from routine biochemical tests were aliquoted and stored at −80 °C until the day of the study. After samples were collected from all groups, the sera were thawed on the day of the experiment. ANXA3 protein levels were analyzed using ELISA with a commercial kit (Elab Science, Wuhan, Hubei, China). In addition, the patients’ complete blood count, routine biochemical, and hormone levels were recorded on the day of sampling. The tests were analyzed using Sysmex XN1000, Roche Cobas e801, and Roche Cobas 8000 analyzer models.

### 2.3. Statistical Analysis

All analyses were two-tailed and performed at an a priori significance level of α = 0.05. Continuous variables were first evaluated for distributional assumptions using the Shapiro–Wilk test. Non-normally distributed data are reported as medians and interquartile ranges (IQR). Categorical data were summarized as counts (percentages). For two-group comparisons (patients with major depression vs. healthy controls), continuous variables that violated normality were compared using the Mann–Whitney U test, and categorical variables were compared using the χ^2^ test or Fisher’s exact test as appropriate. For comparisons across the four BDI severity categories (minimal, mild, moderate, and severe), continuous variables were evaluated using the Kruskal–Wallis test. When the omnibus test was significant, Dunn’s post hoc procedure with Bonferroni adjustment was applied for pairwise comparisons. Where helpful, effect size r was calculated from the Mann–Whitney/Wilcoxon z statistic (Cohen: 0.10 small, 0.30 medium, 0.50 large). The associations between ANXA3 and laboratory parameters were examined using Spearman’s rank correlation (ρ) with 95% confidence intervals (CIs). Heatmaps were used for the visualization.

To identify factors independently associated with group status (MDD vs. control), we fitted a multivariable binary logistic regression model that included the prespecified hematological variables. The model results were reported as odds ratios (ORs) with 95% CIs and Wald *p*-values. To evaluate the predictors of depression severity, we used ordinal logistic regression (proportional odds/PLUM). Model fit was assessed using deviance goodness-of-fit, and the test of parallel lines was examined to verify the proportional odds assumption. The results are expressed as cumulative ORs (Exp [β]), 95% CIs, and *p*-values. Linearity in the logit was inspected for continuous covariates, and multicollinearity was screened using tolerance and variance inflation factor values.

The discriminative ability of the hematological markers was quantified using receiver operating characteristic (ROC) analysis. For the primary comparison (MDD vs. control) and pairwise BDI category contrasts of interest, we reported the area under the curve (AUC) with 95% CIs (DeLong method) and optimal cut-offs selected using Youden’s J. The corresponding sensitivity and specificity (95% confidence intervals [CIs]) were calculated. Multiple ROC analyses were performed across the BDI pairs, and the interpretations emphasized clinically meaningful contrasts to limit multiplicity.

Multivariate pattern analysis. To explore the joint structure among variables across the BDI categories, we performed a principal component analysis (PCA) using MetaboAnalyst (www.metaboanalyst.ca). Prior to PCA, the variables were auto-scaled (mean-centered and unit-variance scaling). We displayed score plots with 95% confidence ellipses, biplots to visualize loadings, and a 3D score plot for PCs 1–3.

Software. Analyses were conducted using IBM SPSS Statistics v23 (IBM Corp., Armonk, NY, USA), MedCalc (Ostend, Belgium) for ROC/AUC CIs and cut-offs, Analyze-it (Analyze-it Software, Leeds, UK) add-in for Excel for supplemental nonparametric and correlation procedures, and MetaboAnalyst for PCA. Graphs were produced using Analyze-it/MedCalc and exported at publication resolution.

The required sample size for the primary between-group comparison (major depression vs. healthy controls) was estimated a priori in G*Power v3.1.9.7 using the Means: Wilcoxon–Mann–Whitney test (two groups) option [18]. We set a two-sided α of 0.05, power (1 – β) of 0.90, and an allocation ratio of 1:1. The anticipated standardized effect size was d = 0.58 (medium–to–large), derived from previous data on group differences in inflammatory indices (pilot or previous studies) [19]. Under these assumptions, the minimum required sample size was 67 per group (total number (n) = 134).

To ensure robust power for secondary analyses (ROC contrasts across BDI categories and multivariable/ordinal models), accommodate potential departures from distributional assumptions, and buffer against any exclusions, we oversampled to n = 90 per group (total n = 180). This exceeded the a priori requirement and thus yielded a power of greater than 90% for the primary endpoint.

For the machine learning analyses, the dataset was randomly divided into training (70%) and test (30%) subsets. Prior to model development, z-score normalization was applied to numerical variables to ensure standardized scaling. Model construction, hyperparameter optimization, and performance evaluation were carried out in Python version 3.12.7 using established libraries, including NumPy [20], Pandas [21], Scikit-Learn [22], and GridSearchCV. All procedures were performed in Jupyter Notebook (version 7.4.5) as the integrated development environment, running on a macOS platform with 16 GB of RAM.

## 3. Results

**Figure 1:**
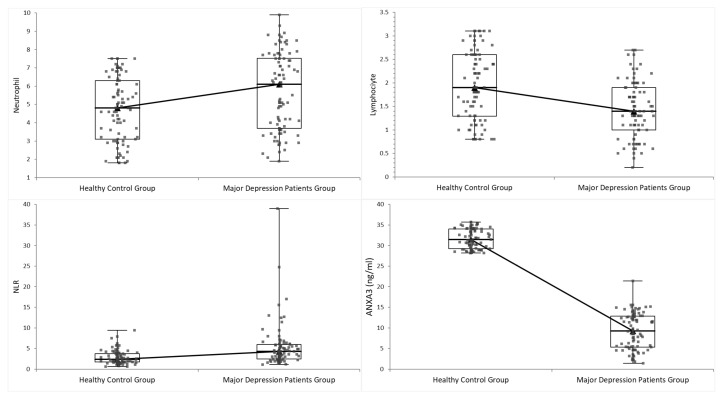
Comparison of neutrophil count, lymphocyte count, neutrophil-to-lymphocyte. ratio (NLR), and serum ANXA3 levels between healthy controls and patients with major depression. Upper left: Neutrophil count (×10^3^/µL). Upper right: Lymphocyte count (×10^3^/µL). Lower left: Neutrophil-to-Lymphocyte Ratio (NLR). Lower right: Serum ANXA3 (ng/mL). X-axis: Groups (Healthy Control, Major Depression); Y-axis: Parameter values as specified.

As summarized in Table 1, the healthy control and major depression (MDD) groups were well-matched for age and sex, with no significant differences in demographic variables. Patients with MDD exhibited a significantly higher neutrophil-to-lymphocyte ratio (NLR) than controls (*p* < 0.001) (Figure 1). This increase in NLR was driven by an inflammatory blood profile, including higher neutrophil counts and lower lymphocyte counts in the MDD group than in healthy individuals (both *p* < 0.005) (Figure 1). Consistent with a proinflammatory state, total white blood cell (WBC) counts were slightly but significantly higher in patients with depression than in controls (*p* < 0.05). In addition to the leukocyte indices, several other hematologic and metabolic differences were observed between the groups (Table 1). Red blood cell (RBC) counts were slightly lower in patients with depression than in controls (*p* = 0.001), whereas platelet counts were significantly higher in the MDD group (*p* = 0.001). Similarly, depressed individuals showed higher fasting glucose levels and higher serum creatinine and alanine aminotransferase (ALT) concentrations than healthy controls (all *p* < 0.001). In contrast, there were no significant group differences in AST (aspartate aminotransferase) or urea levels (both *p* > 0.05). Notably, serum ANXA3 levels were significantly lower in the MDD cohort than in the control group (*p* < 0.001) (Figure 1), representing the most significant difference among all measured parameters (large effect size).

As shown in Table 2, correlation analysis revealed that serum ANXA3 levels were significantly associated with several hematological and biochemical CRC parameters. A statistically significant but weak negative correlation was observed between ANXA3 and total white blood cell (WBC) count, indicating that higher leukocyte counts were modestly associated with lower ANXA3 expression levels. Conversely, a weak positive correlation was observed between ANXA3 expression and red blood cell (RBC) count.

More pronounced associations were observed with inflammatory markers. ANXA3 showed a moderate negative correlation with neutrophil count, platelet count, and the neutrophil-to-lymphocyte ratio (NLR), suggesting that lower ANXA3 levels are associated with higher systemic inflammatory activity. In contrast, the lymphocyte count was positively correlated with ANXA3, further supporting the inverse relationship between ANXA3 expression and inflammatory burden. Additionally, ANXA3 levels were negatively correlated with glucose, alanine aminotransferase (ALT), and creatinine levels, all of which were statistically significant. The association with creatinine was among the strongest observed, suggesting a potential relationship between low ANXA3 levels and impaired renal or metabolic functions. No significant correlation was found between ANXA3 and urea or aspartate aminotransferase (AST) levels. A detailed correlation analysis is presented in Supplementary File (Table S1).

Multivariable logistic regression analysis, summarized in Table 3, was conducted to identify hematological predictors independently associated with the presence of major depression after controlling for potential confounders, including sex and age. The final model included sex, age, neutrophil, lymphocyte, and platelet counts, and the neutrophil-to-lymphocyte ratio (NLR) as independent variables.

Lymphocyte count was significantly inversely associated with major depression status (odds ratio = 0.382, *p* = 0.021), indicating that individuals with lower lymphocyte counts were more likely to be in the major depression group. In contrast, platelet count was positively and significantly associated with major depression (odds ratio = 1.005, *p* = 0.011), suggesting that higher platelet counts increased the odds of depression. The strongest positive predictor in the model was NLR (odds ratio = 1.469, *p* = 0.001), whereby elevated NLR values were associated with substantially higher odds of major depression, even after adjusting for other hematological parameters. There was not statistically significant association between major depression status and Anxa3 levels.

Sex, age, and neutrophil count were not significantly associated with major depression (*p* > 0.05). Although age showed a trend toward significance (*p* = 0.082), its effect size was small (exp (B) = 1.023), and the 95% confidence interval (0.997–1.050) crossed one. Similarly, neutrophil count did not contribute significantly to the model (*p* = 0.274).

**Figure 2:**
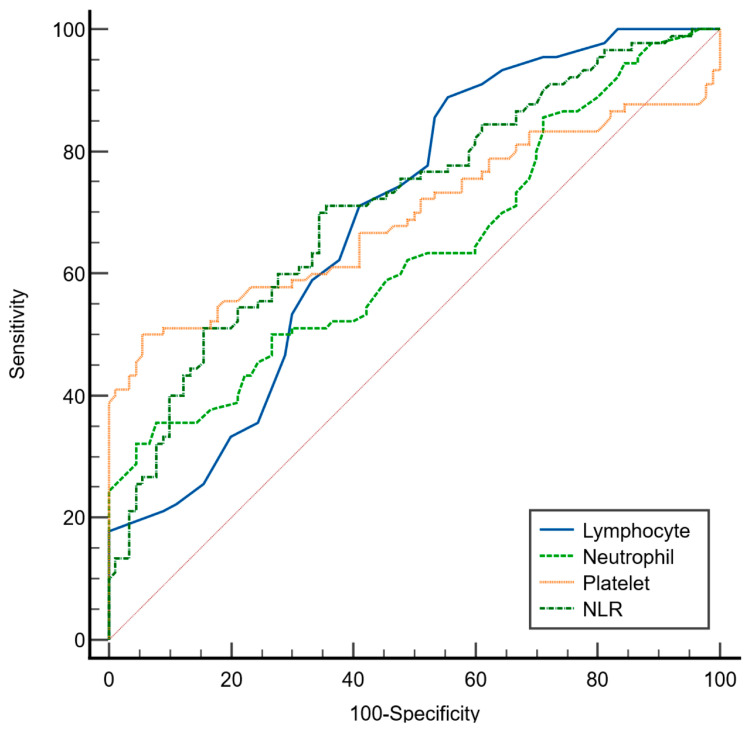
ROC curves for lymphocyte count, neutrophil count, platelet count, and neutrophil-to-lymphocyte ratio (NLR) in differentiating major depression patients from healthy control. X-axis: 1—Specificity. Y-axis: Sensitivity.

As presented in Table 4 and illustrated in Figure 2, ROC curve analysis was performed to evaluate the discriminative ability of the selected hematological parameters in distinguishing patients with major depression from healthy controls using the Youden index. Among the evaluated markers, the neutrophil-to-lymphocyte ratio (NLR) demonstrated the highest area under the curve (AUC = 0.715; 95% CI: 0.643–0.780), indicating a fair discriminatory capacity of NLR. At the optimal cutoff value of >2.73, the NLR yielded a sensitivity of 71% (95% CI: 60–80) and a specificity of 64% (95% CI: 53–74).

The lymphocyte count also showed fair discriminative ability (AUC = 0.692; 95% CI: 0.619–0.759) with a high sensitivity of 88% (95% CI: 80–94) but a relatively low specificity of 44% (95% CI: 34–53) at a cutoff value of ≤2.1 × 10^9^/L, suggesting its potential utility in identifying depressed individuals at the cost of higher false-positive rates. Platelet count presented an AUC of 0.693 (95% CI: 0.621–0.760) with a specificity of 94% (95% CI: 85–98) but lower sensitivity (50%; 95% CI: 39–60) at a cutoff of >373 × 10^9^/L, indicating that it may serve better as a confirmatory rather than a screening marker. Neutrophil count had the lowest discriminative performance (AUC = 0.635; 95% CI: 0.561–0.706) with high specificity (92%; 95% CI: 84–96) but low sensitivity (35%; 95% CI: 25–46) at a cutoff of >7 × 10^9^ cells/L.

**Figure 3:**
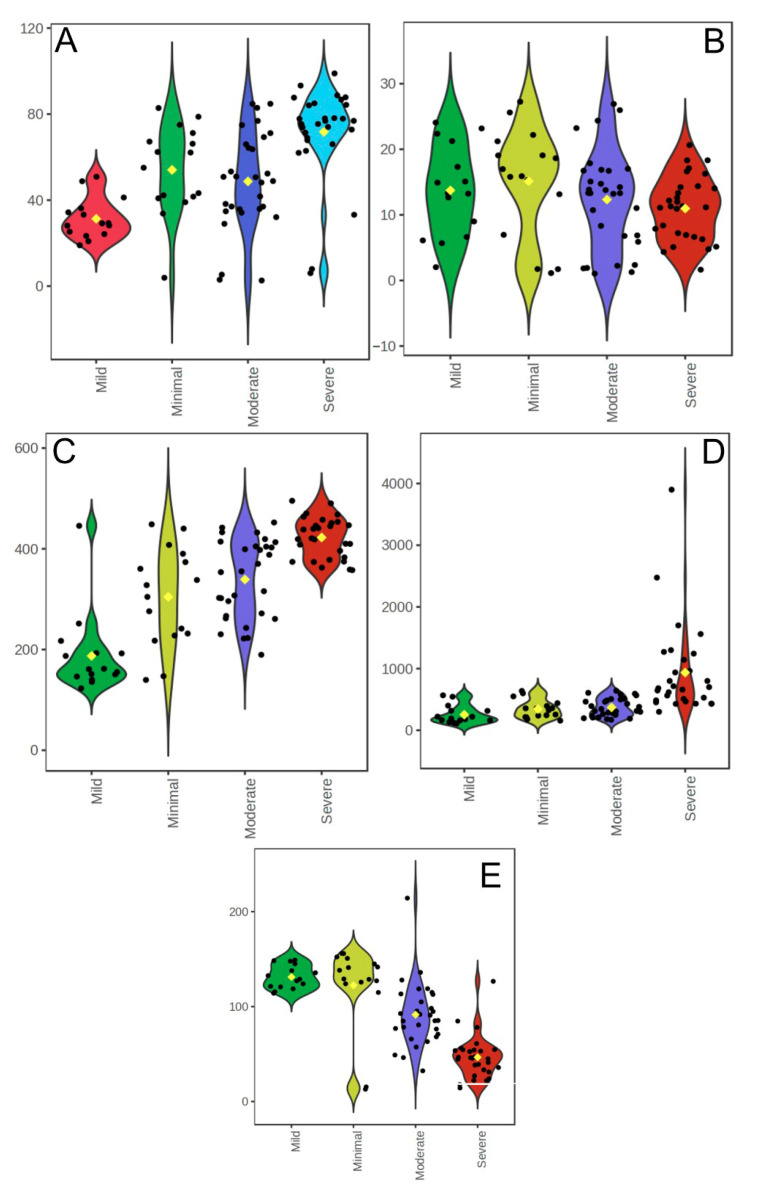
Distribution of hematological and biochemical parameters across BDI severity categories (Minimal, Mild, Moderate, Severe). Violin plots illustrate the distribution of each parameter, with black dots representing individual data points and yellow diamonds indicating group medians. (**A**): Neutrophil count (×10^3^/µL). X-axis: BDI severity categories; Y-axis: Neutrophil count. (**B**): Lymphocyte count (×10^3^/µL). X-axis: BDI severity categories; Y-axis: Lymphocyte count. (**C**): Platelet count (×10^3^/µL). X-axis: BDI severity categories; Y-axis: Platelet count. (**D**): Neutrophil-to-Lymphocyte Ratio (NLR). X-axis: BDI severity categories; Y-axis: NLR. (**E**): Serum ANXA3 (ng/mL). X-axis: BDI severity categories; Y-axis: Serum ANXA3.

A comparison of hematological and biochemical parameters across Beck Depression Inventory (BDI) severity categories revealed several significant differences. WBC counts were higher in the minimal depression group than in the mild depression group (*p* = 0.003) and were significantly elevated in the severe depression group than in the mild (*p* = 0.001) and moderate (*p* = 0.002) depression groups. Neutrophil counts followed a similar pattern, with higher values in the minimal group than in the mild group (*p* = 0.003) and in the severe group compared to both the mild (*p* < 0.001) and moderate (*p* = 0.034) groups. Lymphocyte counts were significantly lower in the mild group than in the moderate (*p* = 0.009) and minimal (*p* = 0.005) groups and in the severe group than in both the mild (*p* < 0.001) and moderate (*p* = 0.024) groups. Platelet counts were significantly higher in the severe group than in the moderate group (*p* = 0.010) (Table 5 and Figure 3).

The NLR was higher in the minimal group than in the mild group (*p* = 0.002) and in the severe group than in the mild (*p* < 0.001), moderate (*p* < 0.001), and minimal (*p* < 0.001) groups (Figure 3D). Glucose levels were significantly higher in the mild group than in the moderate (*p* = 0.002) and severe (*p* = 0.004) groups. Serum ANXA3 concentrations were markedly reduced in the moderate (*p* = 0.001) and severe (*p* = 0.001) groups compared to the minimal group and were significantly lower in the severe group than in the mild (*p* = 0.001) and moderate groups (*p* = 0.001) (Figure 3E). No statistically significant differences were observed between the groups in terms of age, RBC count, urea, creatinine, ALT, or AST levels (*p* > 0.05).

Overall, these results indicate that higher depression severity is associated with elevated WBC, neutrophil, and platelet counts, NLR, and glucose levels, and reduced lymphocyte and ANXA3 levels. This pattern supports a link between an increased depressive symptom burden and a systemic inflammatory response.

Ordinal logistic regression analysis identified ANXA3, NLR, and glucose levels as significant predictors of higher BDI categories (Table 6). Each one-unit increase in NLR was associated with a 1.35-fold greater likelihood of being in a higher depression severity category (*p* = 0.011). Similarly, lower glucose (OR = 0.91, *p* = 0.005) and reduced ANXA3 concentrations (OR = 0.50, *p* < 0.001) independently predicted greater depression severity. In contrast, gender, age, and platelet count were not significant predictors (*p* > 0.05).

**Figure 4:**
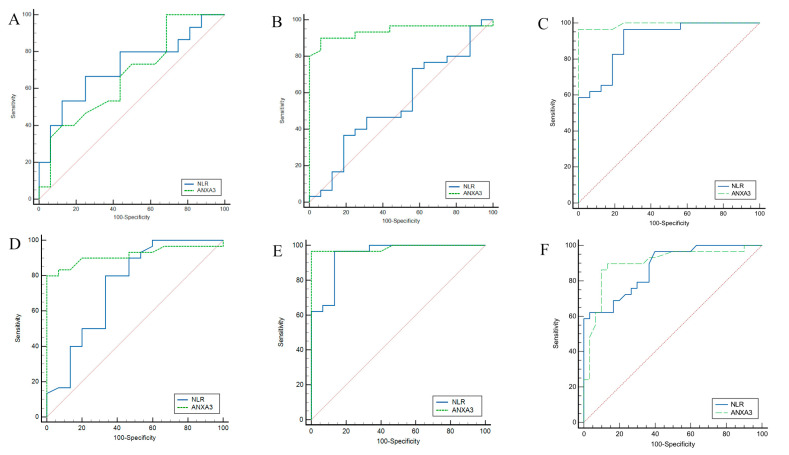
Receiver operating characteristic (ROC) curves for NLR and ANXA3 in pairwise discrimination between BDI severity categories: (**A**) Minimal vs. Mild, (**B**) Minimal vs. Moderate, (**C**) Minimal vs. Severe, (**D**) Mild vs. Moderate, (**E**) Mild vs. Severe, and (**F**) Moderate vs. Severe.

Pairwise ROC analysis was performed to evaluate the ability of NLR and serum ANXA3 levels to discriminate between different Beck Depression Inventory (BDI) severity categories (Table 7 and Figure 4). For minimal–mild discrimination, NLR at a cutoff value of ≤2.22 yielded an AUC of 0.717 (95% CI: 0.527-0.863), with 67% sensitivity and 75% specificity, whereas serum ANXA3 at a cutoff of ≤14.9 demonstrated an AUC of 0.671 (95% CI: 0.480–0.821) with higher sensitivity (100%) but lower specificity (31%). For minimal–moderate discrimination, NLR showed limited discriminative ability (AUC: 0.548), whereas serum ANXA3 performed markedly better (AUC: 0.939; 95% CI: 0.826–0.988), achieving 90% sensitivity and 93% specificity, respectively. For minimal–severe discrimination, NLR achieved an AUC of 0.907 (95% CI: 0.783–0.973), whereas serum ANXA3 reached near-perfect accuracy (AUC: 0.992; 95% CI: 0.907–1.00), with both sensitivity and specificity exceeding 96%. When discriminating between mild and moderate depression, NLR (cutoff > 2.22) had an AUC of 0.744 (95% CI: 0.593–0.863), while serum ANXA3 (cutoff ≤ 11.3) had an AUC of 0.917 (95% CI: 0.795–0.978) with perfect (100%) specificity. For mild–severe discrimination, both NLR and serum ANXA3 achieved high accuracy (AUC: 0.945 and 0.985, respectively). For moderate–severe discrimination, NLR (cutoff > 6.07) showed an AUC of 0.875, while serum ANXA3 (cutoff ≤ 6.1) demonstrated a slightly lower AUC of 0.897 but maintained a high sensitivity (89%) and specificity (86%) compared to NLR.

**Figure 5:**
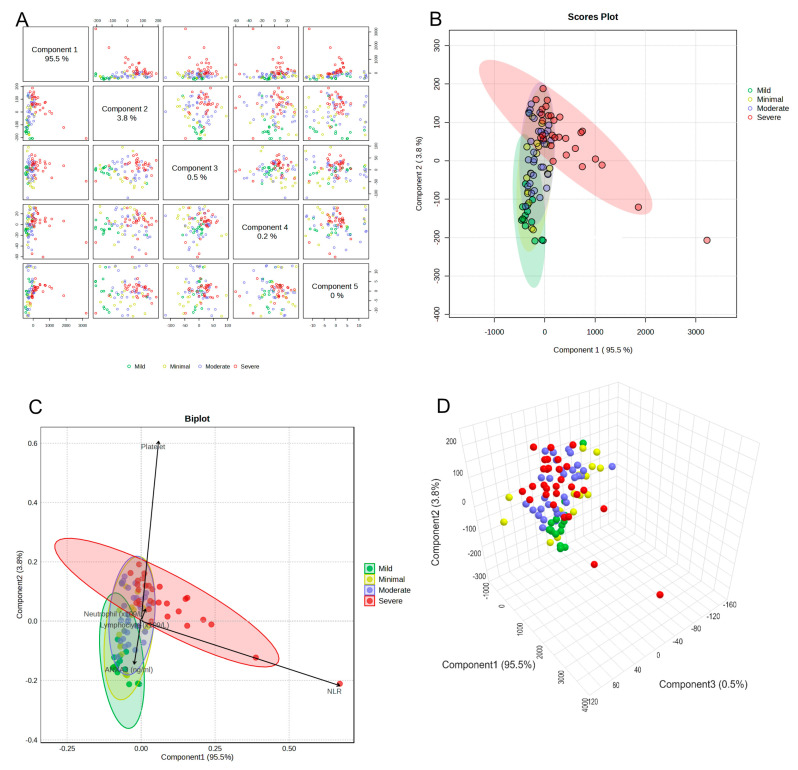
Principal component analysis (PCA) of hematological parameters in different Beck Depression Inventory (BDI) severity groups: (**A**) Pairwise component scatter plots, (**B**) 2D scores plot with 95% confidence ellipses, (**C**) biplot showing variable loadings, and (**D**) 3D scores plot.

Principal component analysis (PCA) was performed to explore the overall distribution and separation of participants according to hematological parameters across the BDI severity groups. As shown in Figure 5A, the pairwise scatter plots for the first five components indicated that the majority of the variance was explained by the first principal component (PC1: 95.5%), followed by PC2 (3.8%), whereas the remaining components contributed minimally to the variance. The 2D score plot (Figure 5B) demonstrated a partial separation between the Severe and Mild groups, with the Minimal and Moderate groups showing greater overlap, as depicted by the 95% confidence ellipses.

The biplot (Figure 5C) revealed that the neutrophil-to-lymphocyte ratio (NLR) was the strongest contributor to PC1, oriented in the positive direction, whereas platelet count and neutrophil levels showed moderate contributions along PC2. Age and glucose levels exhibited smaller loadings, indicating a weaker influence on the separation of the groups in the PCA space. Notably, the Severe group clustered predominantly toward the positive PC1 axis, consistent with higher NLR values, whereas the mild group was positioned toward the negative PC1 axis.

The 3D score plot (Figure 5D) provided an enhanced visualization of group clustering, highlighting a clearer separation of the severe group from the others when incorporating PC3 (0.5% variance explained). These findings suggest that the NLR, followed by platelet and neutrophil counts, is the most influential variable in differentiating depression severity categories in the multivariate space.

**Figure 6:**
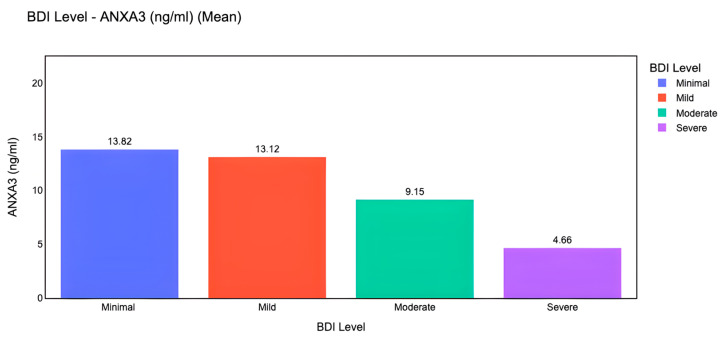
Mean serum ANXA3 (ng/mL) levels across BDI severity categories (Minimal, Mild, Moderate, Severe). Bars represent group means, showing a progressive decrease in ANXA3 levels with increasing depression severity.

**Figure 7:**
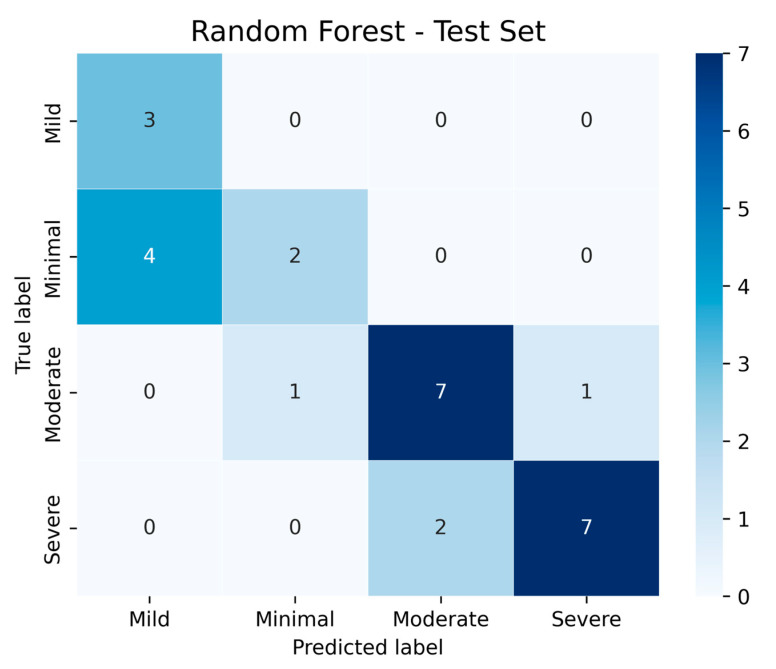
Confusion matrix of the Random Forest model (70/30 train–test split, z-score normalization) for classifying depression severity. The model achieved higher accuracy in identifying moderate and severe cases, with some overlap between minimal and mild categories.

In addition to classical statistical analyses, we implemented machine learning models to evaluate the predictive capacity of ANXA3 and related biomarkers. Machine learning-based visualization further demonstrated that mean serum ANXA3 levels decreased progressively across depression severity categories, with the highest levels in the minimal and mild groups and the lowest levels in the severe group (Figure 6). A Random Forest classifier was trained using a 70/30 train–test split with z-score normalization. On the test set, the confusion matrix (Figure 7) showed that moderate and severe depression categories were classified with relatively high accuracy, whereas some overlap was observed between minimal and mild categories. These results suggest that machine learning approaches can complement traditional analyses by improving discrimination between clinically meaningful severity levels.

## 4. Discussion

In our study, we demonstrated that serum ANXA3 levels were significantly lower in patients with major depressive disorder than in healthy controls and that ANXA3 levels were negatively correlated with the neutrophil-to-lymphocyte ratio (NLR). Our findings suggest that ANXA3 may play an important role in the pathophysiology of depression and may serve as a potential biomarker, particularly for inflammation-related disorders in women.

The robustness of our statistical model suggests that ANXA3 may have potential clinical significance in classifying depression severity. Serum ANXA3 levels consistently showed higher AUC values than NLR across all comparisons, demonstrating near-perfect performance, particularly in distinguishing moderate from severe MDD. To facilitate interpretation, we have also reformatted the ordinal regression outputs into a concise summary table, which highlights the strong predictive role of ANXA3 and NLR in relation to depression severity.

We demonstrated that NLR and lymphocyte count are highly discriminatory and may serve as potential indicators of systemic inflammation in patients with this condition. Our regression model demonstrated that hematologic parameters, specifically decreased lymphocyte count, increased platelet count, and elevated NLR, were independently associated with the presence of major depression, supporting the role of inflammation in the pathophysiology of MDD.

The integration of a machine learning model adds translational value to our findings. The Random Forest classifier demonstrated that ANXA3, combined with inflammatory markers such as NLR, can reliably differentiate moderate and severe MDD cases. This highlights the potential of ML to capture complex patterns in biomarker data that may not be evident with conventional statistics. Although our dataset size limits generalization, these results illustrate how explainable ML models can enhance early diagnosis and severity stratification in psychiatric disorders. Future large-scale studies are needed to validate and refine these predictive frameworks.

Our findings are consistent with those of previous studies investigating ANXA3 alterations in patients with psychiatric disorders. Öztürk et al. [23] reported significantly lower ANXA3 levels in both schizophrenia and bipolar disorder patients compared with healthy controls, emphasizing that the most pronounced decreases were in schizophrenia patients. Similarly, Joaquim et al. [24] found significantly lower ANXA3 levels in medication-naïve, first-episode schizophrenia patients compared to patients with bipolar disorder and healthy controls. Considering these data and studies, it is clear that ANXA3 deficiency may be associated not only with psychotic disorders but also with several psychiatric conditions. Dysregulation of phospholipase A2 (PLA2) activity, which is closely linked to annexin family proteins, has been consistently reported in patients with psychotic disorders [25,26]. Gheorghe et al. A recent study by Carbone et al. on patients with depression and anxiety highlighted the high prevalence of depression and anxiety in primary hyperparathyroidism and reported significant improvements in psychological symptoms after parathyroidectomy [27]. Similarly, Carbone et al. showed that vitamin D deficiency and elevated parathyroid hormone (PTH) levels were associated with increased anxiety severity and poor sleep quality in patients with generalized anxiety disorder [28]. These results suggest a possible link between calcium-PTH homeostasis and psychiatric symptoms that may intersect with ANXA3-related pathways.

Furthermore, the dual role of T-type calcium channels in anxiety has been described by Zhao et al. [29]. In this case, overactivation or suppression of these channels may regulate anxiety-related behaviors, suggesting that calcium homeostasis and membrane signaling proteins, such as ANXA3, may have evolved through similar neurobiological mechanisms.

In our study, the significantly lower serum ANXA3 levels in patients with depression suggest that the calcium-binding annexin family may play a role in the pathophysiology of depression. Similarly to our findings, Zhang et al. demonstrated that deletion of ITPR2, an endoplasmic reticulum calcium channel in oligodendrocytes, disrupts calcium homeostasis, inhibits myelination, and leads to depression-like behavior [30]. Both studies support the strong association between impaired calcium signaling and depressive phenotypes. Wang et al. reported that suppressing mitochondrial calcium uniporter (MCU) in an animal model of Alzheimer’s disease reduced anxiety and depressive behavior in hippocampal neurons [31]. These findings suggest that both peripheral calcium-binding protein levels and central mitochondrial calcium balance play important roles in the mechanisms of depression.

A key challenge of this study is that the decreased ANXA3 levels in MDD parallel the findings in schizophrenia and bipolar disorder, suggesting its potential as a cross-diagnostic biomarker for these disorders. More importantly, the observed negative correlation between ANXA3 and NLR supports the hypothesis that inflammatory processes may be an important mechanistic link. Future research should examine whether ANXA3 alterations in MDD are state or trait markers, their response to pharmacological and non-pharmacological interventions, and their possible interaction with dysregulation of the calcium-PTH-vitamin D axis. In addition to conventional statistical methods, future studies should incorporate artificial intelligence and machine learning approaches. Explainable models, such as random forests combined with SHAP analysis, may improve the early diagnostic potential of ANXA3 while maintaining transparency regarding the relative contributions of individual biomarkers. This integrative approach could strengthen the translational impact of our findings and support their clinical relevance.

### Limitations

Our study has several limitations. First, our research focused solely on protein-level analyses of circulating ANXA3 measured by ELISA, as this approach provides direct clinical applicability. However, other omics layers, particularly transcriptomics, were not integrated. Assessing ANXA3 expression at the mRNA level in future studies will be important to determine whether the observed protein-level decreases are also reflected at the transcriptional level, thus strengthening the biological plausibility of ANXA3 as a biomarker of depression severity. Second, data on patients’ medication status, body mass index (BMI), smoking habits, and other lifestyle factors were not systematically collected. These variables can influence both ANXA3 concentrations and inflammatory markers, and their absence limits our ability to fully account for potential confounding factors. Future studies should include these parameters to better determine the independent contribution of ANXA3 to major depressive disorder. Despite these limitations, the current findings provide new clinical evidence supporting a potential role of ANXA3 in MDD, while also laying the groundwork for future multi-omics and more extensive controlled studies.

## 5. Conclusions

Our study showed that serum ANXA3 levels were significantly decreased in patients with major depressive disorder and were strongly associated with systemic inflammatory markers and severity of depression. ANXA3 may serve as a potential biomarker for identifying and monitoring the severity of depression. More comprehensive studies are needed to confirm our findings and investigate the role of ANXA3 in the pathophysiology of depression.

## Figures and Tables

**Table 1:** Comparison of demographic, hematological, and biochemical parameters between healthy controls and patients with major depression.

		Healthy Control Group (N = 90)	Major Depression Patients Group (N = 90)	Effect Size	*p* Value
**Gender**	**Female**	42 (47)	48 (53)		0.958
	**Male**	48 (53)	42 (47)		
**BDI**	**Minimal**	0 (0)	16 (18)		N.D.
	**Mild**	0 (0)	15 (17)		
	**Moderate**	0 (0)	30 (33)		
	**Severe**	0 (0)	29 (32)		
**Age (year)**		42 (31–52)	45.5 (33–57)	r = −0.138	0.064
**WBC (×10^9^/L)**		7.3 (5.8–9)	8.2 (6.2–9.7)	r = −0.179	**0.016**
**RBC (×10^12^/L)**		4.9 (4.5–5.5)	4.6 (4.1–5.1)	r = −0.301	**0.001**
**Platelet (×10^9^/L)**		268 (200–350)	372 (242–419)	r = −0.334	**0.001**
**Glucose (mg/dL)**		85 (80–93)	95 (87–102)	r = −0.44	**0.001**
**Urea (mg/dL)**		31.5 (25–42)	34 (25–42)	r = −0.044	**0.556**
**Creatinine (mg/dL)**		0.9 (0.7–1)	1.3 (1.1–1.5)	r = −0.674	**0.001**
**ALT (U/L)**		25 (17–37)	39 (23–51)	r = −0.314	**0.001**
**AST (U/L)**		26.5 (16–37)	26 (12–37)	r = −0.035	0.636
**ANXA3 (ng/mL)**		31.5 (29.2–34)	9.25 (5.3–12.8)	r = −0.864	**0.001**
**Neutrophil (×10^9^/L**		4.8 (3.1–6.3)	6.1 (3.7–7.5)	r = −0.234	**0.002**
**Lymphocyte (×10^9^/L)**		1.9 (1.3–2.6)	1.4 (1–1.9)	r = −0.333	**0.001**
**NLR**		2.41 (1.7–3.75)	4.29 (2.44–5.93)	r = −0.371	**0.001**

NLR = neutrophil-to-lymphocyte ratio; WBC = white blood cell count; RBC = red blood cell count; BDI = Beck Depression Inventory; ALT = alanine aminotransferase; AST = aspartate aminotransferase; ANXA3 = Annexin A3.

**Table 2:** Correlation analysis between serum ANXA3 concentrations and hematological/biochemical parameters in the study population.

	Age	WBC	RBC	Neutrophil	Lymphocyte	Platelet	NLR	Glucose	Urea	ALT	Creatinine	AST	ANXA3
Age	−	0.017	−0.153	−0.008	−0.035	0.016	0.015	0.096	0.016	−0.064	0.047	0.045	−0.151
WBC	0.017	−	−0.101	0.469	0.021	0.227	0.261	0.039	−0.187	0.029	0.086	−0.059	−0.301
RBC	−0.153	−0.101	−	−0.015	0.120	−0.047	−0.082	−0.118	0.000	−0.089	−0.092	0.017	0.274
Neutrophil	−0.008	0.469	−0.015	−	−0.066	0.399	0.686	−0.036	−0.075	0.074	0.132	0.050	−0.316
Lymphocyte	−0.035	0.021	0.120	−0.066	−	−0.223	−0.735	−0.145	−0.033	−0.020	−0.186	−0.031	0.407
Platelet	0.016	0.227	−0.047	0.399	−0.223	−	0.386	0.141	0.145	0.031	0.230	−0.026	−0.459
NLR	0.015	0.261	−0.082	0.686	−0.735	0.386	−	0.075	−0.040	0.068	0.199	0.078	−0.467
Glucose	0.096	0.039	−0.118	−0.036	−0.145	0.141	0.075	−	0.054	0.147	0.328	0.019	−0.322
Urea	0.016	−0.187	0.000	−0.075	−0.033	0.145	−0.040	0.054	−	−0.066	0.030	−0.037	−0.040
ALT	−0.064	0.029	−0.089	0.074	−0.020	0.031	0.068	0.147	−0.066	−	0.179	0.025	−0.250
Creatinine	0.047	0.086	−0.092	0.132	−0.186	0.230	0.199	0.328	0.030	0.179	−	−0.061	−0.535
AST	0.045	−0.059	0.017	0.050	−0.031	−0.026	0.078	0.019	−0.037	0.025	−0.061	−	0.052
ANXA3	−0.151	−0.301	0.274	−0.316	0.407	−0.459	−0.467	−0.322	−0.040	−0.250	−0.535	0.052	−

Red color indicates negative correlation; blue color indicates positive correlation. Color intensity is linearly related to the correlation level.

**Table 3:** Logistic regression analysis of hematological parameters associated with major depression.

	B	Wald	Sig.	Odd Ratio Exp(B)	95% CI for EXP(B)	
					Lower	Upper
**Gender**	−0.038	0.012	0.911	0.963	0.492	1.884
**Age (year)**	0.023	3.020	0.082	1.023	0.997	1.050
**Neutrophil (×10^9^/L)**	0.157	1.195	0.274	1.170	0.883	1.550
**Lymphocyte (×10^9^/L)**	−0.961	5.327	**0.021**	0.382	0.169	0.865
**Platelet (×10^9^/L)**	0.005	6.409	**0.011**	1.005	1.001	1.009
**Serum ANXA3**	−4.436	0.001	0.99	0.012	0.001	1.67
**NLR**	0.385	17.992	**0.00017**	1.469	1.23	1.755

**Table 4:** The predictive value results of hematological parameters for discriminating patients with major depression from healthy controls.

Variable	Cutoff	AUC (95% CI)	Sensitivity (95% CI)	Specificity (95% CI)
**Lymphocyte**	≤2.1	0.692 (0.619–0.759)	88 (80–94)	44 (34–53)
**Neutrophil**	>7	0.635 (0.561–0.706)	35 (25–46)	92 (84–96)
**Platelet**	>373	0.693 (0.621–0.760)	50 (39–60)	94 (85–98)
**NLR**	>2.73	0.715 (0.643–0.780)	71 (60–80)	64 (53–74)

CI: Confidence intervals.

**Table 5:** Comparison of demographic, hematological, and biochemical parameters across different Beck Depression Inventory (BDI) severity categories.

		Minimal (N = 16)	Mild (N = 15)	Moderate (N = 30)	Severe (N = 29)
**Gender**	**Female**	12 (75)	5 (33)	17 (57)	14 (48)
	**Male**	4 (25)	10 (67)	13 (43)	15 (52)
**Age (year)**		48.5 (36–56.5) ^a^	50 (39–57) ^a^	40.5 (29–57) ^a^	44 (37–55) ^a^
**WBC (×10^9^/L)**		8.3 (6.6–9.75) ^a^	5.3 (4.6–6.2) ^b^	7.55 (5.7–8.7) ^b^	9.4 (9.1–10.4) ^a^
**RBC (×10^12^/L)**		4.3 (3.85–4.65) ^a^	4.4 (4–5.2) ^a^	4.7 (4.1–5.1) ^a^	4.8 (4.3–5.1) ^a^
**Neutrophil (×10^9^/L)**		5.85 (4.15–6.9) ^a^	2.9 (2.4–3.6) ^b^	5.05 (3.6–6.6) ^a^	7.7 (7.1–8.4) ^c^
**Lymphocyte (×10^9^/L)**		1.9 (1.55–2.05) ^a^	1.5 (0.9–2.1) ^a^	1.45 (1.1–2) ^a^	1.1 (0.7–1.4) ^b^
**Platelet (×10^9^/L)**		316.5 (230–382) ^a^	161 (146–193) ^a^	354.5 (267–405) ^a^	421 (383–451) ^c^
**Glucose (mg/dL)**		101.5 (96–106) ^a^	97 (90–104) ^a^	90 (86–98) ^b^	94 (87–100) ^b^
**Urea (mg/dL)**		31.5 (26.5–42.5) ^a^	33 (23–44) ^a^	40 (32–43) ^a^	31 (23–41) ^a^
**Creatinine (mg/dL)**		1.4 (1.3–1.5) ^a^	1.3 (1–1.5) ^a^	1.3 (1.1–1.5) ^a^	1.2 (1.1–1.4) ^a^
**ALT (U/L)**		41.5 (16.5–54) ^a^	41 (32–53) ^a^	38 (25–53) ^a^	29 (23–47) ^a^
**AST (U/L)**		27.5 (18.5–37.5) ^a^	28 (16–37) ^a^	18 (9–36) ^a^	28 (13–38) ^a^
**ANXA3 (ng/mL)**		13.9 (12.8–15.1) ^a^	12.9 (12.1–14.5) ^a^	8.8 (7.1–11.3) ^b^	4.6 (3.3–5.4) ^c^
**NLR**		3.41 (2.27–4.17) ^a^	1.92 (1.6–3.17) ^a^	3.29 (2.5–5) ^a^	6.82 (4.86–11.4) ^b^

^a,b,c^: Different letters in the same row indicated statistically significant *p* < 0.05.

**Table 6:** Ordinal logistic regression predicting higher BDI category.

Predictor	β (SE)	OR (95% CI)	*p*-Value
Gender (Male vs. Female)	−0.77 (0.41)	0.46 (0.16–1.35)	0.157
Age	−0.02 (0.02)	0.98 (0.94–1.01)	0.272
NLR	0.30 (0.12)	1.35 (1.12–1.85)	0.011
Platelet	0.00 (0.00)	1.00 (0.99–1.01)	0.588
Glucose	−0.09 (0.03)	0.91 (0.85–0.97)	0.005
ANXA3	−0.69 (0.13)	0.50 (0.39–0.65)	0.00002

**Table 7:** Pairwise ROC analysis results for NLR and ANXA3 in discriminating between BDI severity categories.

	**Minimal–Mild Discrimination**			
**Variable**	**Cutoff**	**AUC (95% CI)**	**Sensitivity (95% CI)**	**Specificity (95% CI)**
**NLR**	≤2.22	0.717 (0.527–0.863)	67 (38–88)	75 (48–92)
**Serum ANXA3**	≤14.9	0.671 (0.48–0.821)	100 (78–100)	31 (11–58)
	**Minimal–Moderate discrimination**			
**NLR**	>4.4	0.548 (0.394–0.695)	36 (20–56)	82 (54–94)
**Serum ANXA3**	≤11.9	0.939 (0.826–0.988)	90 (73–97)	93 (69–100)
	**Minimal–Severe discrimination**			
**NLR**	>3.94	0.907 (0.783–0.973)	95 (82–99)	75 (47–92)
**Serum ANXA3**	≤8.5	0.992 (0.907–1.00)	96 (82–99)	100 (79–100)
	**Mild–Moderate discrimination**			
**NLR**	>2.22	0.744 (0.593–0.863)	80 (61–92)	67 (38–88)
**Serum ANXA3**	≤11.3	0.917 (0.795–0.978)	80 (61–92)	100 (78–100)
	**Mild–Severe discrimination**			
**NLR**	>4	0.945 (0.831–0.991)	96 (82–99)	87 (59–98)
**Serum ANXA3**	≤8.5	0.985 (0.893–1.00)	96 (82–99)	100 (78–100)
	**Moderate–Severe discrimination**			
**NLR**	>6.07	0.875 (0.763–0.947)	62 (42–79)	96 (82–99)
**Serum ANXA3**	≤6.1	0.897 (0.789–0.961)	89 (72–97)	86 (69–96)

## Data Availability

The data presented in this study are available on request from the corresponding author.

## Supplementary Materials

The following supporting information can be downloaded at: https://www.mdpi.com/article/10.3390/life15091456/s1, Table S1: The correlation analysis of all variables.
