# Supplementary material for: Lower ANXA3 Levels May Be Related to Major Depressive Disorder
Source: Life (Basel). 2025 Sep 17;15(9):1456. doi: 10.3390/life15091456 (PMC12471239; doi:10.3390/life15091456)
Supplement: Supplementary file 1 [file life-15-01456-s001.zip › life-3872190-supplementary.pdf]

Table S1: The correlation analysis of all variables.

			Age (year)	WBC (x109/L)	RBC (x1012/L)	Neutrophil (x109/L	Lymphocyte (x109/L)	Platelet (x109/L)	NLR	Glucose (mg/dL)	Urea (mg/dL)	Creatinine (mg/dL)	ALT (U/L)	AST (U/L)	ANXA3 (ng/ml)
Spearman's rho	Age (year)	Correlation Coefficient	1,000	,017	-,153*	-,008	-,035	,016	,015	,096	,016	,047	-,064	,045	-,151*
		Sig. (2-tailed)	.	,817	,041	,916	,643	,830	,841	,198	,834	,534	,392	,550	,043
		N	180	180	180	180	180	180	180	180	180	180	180	180	180
	WBC (x109/L)	Correlation Coefficient	,017	1,000	-,101	,469**	,021	,227**	,261**	,039	-,187*	,086	,029	-,059	-,301**
		Sig. (2-tailed)	,817	.	,175	,000	,775	,002	,000	,605	,012	,251	,700	,435	,000
		N	180	180	180	180	180	180	180	180	180	180	180	180	180
	RBC (x1012/L)	Correlation Coefficient	-,153*	-,101	1,000	-,015	,120	-,047	-,082	-,118	,000	-,092	-,089	,017	,274**
		Sig. (2-tailed)	,041	,175	.	,839	,109	,532	,273	,114	,997	,220	,234	,826	,000
		N	180	180	180	180	180	180	180	180	180	180	180	180	180
	Neutrophil (x109/L	Correlation Coefficient	-,008	,469**	-,015	1,000	-,066	,399**	,686**	-,036	-,075	,132	,074	,050	-,316**
		Sig. (2-tailed)	,916	,000	,839	.	,380	,000	,000	,632	,317	,076	,320	,505	,000
		N	180	180	180	180	180	180	180	180	180	180	180	180	180
	Lymphocyte (x109/L)	Correlation Coefficient	-,035	,021	,120	-,066	1,000	-,223**	-,735**	-,145	-,033	-,186*	-,020	-,031	,407**
		Sig. (2-tailed)	,643	,775	,109	,380	.	,003	,000	,053	,662	,012	,788	,676	,000
		N	180	180	180	180	180	180	180	180	180	180	180	180	180
	Platelet (x109/L)	Correlation Coefficient	,016	,227**	-,047	,399**	-,223**	1,000	,386**	,141	,145	,230**	,031	-,026	-,459**
		Sig. (2-tailed)	,830	,002	,532	,000	,003	.	,000	,059	,051	,002	,682	,725	,000
		N	180	180	180	180	180	180	180	180	180	180	180	180	180
	NLR	Correlation Coefficient	,015	,261**	-,082	,686**	-,735**	,386**	1,000	,075	-,040	,199**	,068	,078	-,467**
		Sig. (2-tailed)	,841	,000	,273	,000	,000	,000	.	,320	,597	,007	,366	,295	,000
		N	180	180	180	180	180	180	180	180	180	180	180	180	180
	Glucose (mg/dL)	Correlation Coefficient	,096	,039	-,118	-,036	-,145	,141	,075	1,000	,054	,328**	,147*	,019	-,322**
		Sig. (2-tailed)	,198	,605	,114	,632	,053	,059	,320	.	,473	,000	,049	,796	,000
		N	180	180	180	180	180	180	180	180	180	180	180	180	180
	Urea (mg/dL)	Correlation Coefficient	,016	-,187*	,000	-,075	-,033	,145	-,040	,054	1,000	,030	-,066	-,037	-,040
		Sig. (2-tailed)	,834	,012	,997	,317	,662	,051	,597	,473	.	,687	,378	,618	,594
		N	180	180	180	180	180	180	180	180	180	180	180	180	180
	Creatinine (mg/dL)	Correlation Coefficient	,047	,086	-,092	,132	-,186*	,230**	,199**	,328**	,030	1,000	,179*	-,061	-,535**
		Sig. (2-tailed)	,534	,251	,220	,076	,012	,002	,007	,000	,687	.	,016	,417	,000
		N	180	180	180	180	180	180	180	180	180	180	180	180	180
	ALT (U/L)	Correlation Coefficient	-,064	,029	-,089	,074	-,020	,031	,068	,147*	-,066	,179*	1,000	,025	-,250**
		Sig. (2-tailed)	,392	,700	,234	,320	,788	,682	,366	,049	,378	,016	.	,743	,001
		N	180	180	180	180	180	180	180	180	180	180	180	180	180
	AST (U/L)	Correlation Coefficient	,045	-,059	,017	,050	-,031	-,026	,078	,019	-,037	-,061	,025	1,000	,052
		Sig. (2-tailed)	,550	,435	,826	,505	,676	,725	,295	,796	,618	,417	,743	.	,489
		N	180	180	180	180	180	180	180	180	180	180	180	180	180

	ANXA3 (ng/ml)	Correlation Coefficient	-,151*	-,301**	,274**	-,316**	,407**	-,459**	-,467**	-,322**	-,040	-,535**	-,250**	,052	1,000
		Sig. (2-tailed)	,043	,000	,000	,000	,000	,000	,000	,000	,594	,000	,001	,489	.
		N	180	180	180	180	180	180	180	180	180	180	180	180	180
